# Antiplatelet Activity of Isorhamnetin via Mitochondrial Regulation

**DOI:** 10.3390/antiox10050666

**Published:** 2021-04-25

**Authors:** Lyanne Rodríguez, Lina Badimon, Diego Méndez, Teresa Padró, Gemma Vilahur, Esther Peña, Basilio Carrasco, Hermine Vogel, Iván Palomo, Eduardo Fuentes

**Affiliations:** 1 Thrombosis Research Center, Department of Clinical Biochemistry and Immunohaematology, Faculty of Health Sciences, Medical Technology School, Universidad de Talca, Talca 3460000, Chile; lyannerodriguez89@gmail.com (L.R.); dmendez12@alumnos.utalca.cl (D.M.); 2 Cardiovascular Program—ICCC and CiberCV, IR-Hospital de la Santa Creu i Sant Pau, 08025 Barcelona, Spain; lbadimon@santpau.cat (L.B.); tpadro@santpau.cat (T.P.); gvilahur@santpau.cat (G.V.); epena@santpau.cat (E.P.); 3 Centro de Estudios en Alimentos Procesados, Talca 3460000, Chile; bcarrasco@ceap.cl; 4 CENATIV, Departamento de Horticultura, Facultad de Ciencias Agrarias, Universidad de Talca, Talca 3460000, Chile; hvogel@utalca.cl

**Keywords:** isorhamnetin, flavonoids, antithrombotic, antiplatelet, foods

## Abstract

With the diet, we ingest nutrients capable of modulating platelet function, which plays a crucial role in developing cardiovascular events, one of the leading causes of mortality worldwide. Studies that demonstrate the antiplatelet and antithrombotic potential of bioactive compounds are vital to maintaining good cardiovascular health. In this work, we evaluate the flavonol isorhamnetin’s antiplatelet effect on human platelets, using collagen, thrombin receptor activator peptide 6 (TRAP-6), and phorbol myristate acetate (PMA) as agonists. Isorhamnetin induced a significant inhibition on collagen- and TRAP-6-induced platelet aggregation, with half-maximum inhibitory concentration (IC_50_) values of 8.1 ± 2.6 and 16.1 ± 11.1 µM, respectively; while it did not show cytotoxic effect. Isorhamnetin reduced adenosine triphosphate levels (ATP) in platelets stimulated by collagen and TRAP-6. We also evidenced that isorhamnetin’s antiplatelet activity was related to the inhibition of mitochondrial function without effect on reactive oxygen species (ROS) levels. Additionally, we investigated isorhamnetin’s effect on thrombus formation in vitro under flow conditions on the damaged vessel wall. In this context, we demonstrate that isorhamnetin at 20 µM induced a significant inhibition on platelet deposition, confirming its antithrombotic effect. Our findings corroborate the antiplatelet and antithrombotic potential of isorhamnetin present in many foods of daily consumption.

## 1. Introduction

The number of deaths from diet-related cardiovascular events increased between 2010 and 2016 [1]. With a healthy diet, rich mainly in fruits and vegetables, more than 30% of deaths could be prevented [2,3]. A balanced diet is vital to avoid premature death [1]. It is necessary to promote the concept of a healthy diet, a diet that includes the intake of minimally processed foods and it is rich in bioactive products present in fruits, beans, fish, seeds, whole grains, yogurt, nuts, vegetable oils, and vegetables [3]. Indeed, numerous investigations indicate that the dietary intake of flavonoids, such as quercetin and its derivatives present in fruits and vegetables, can reduce the risk of cardiovascular diseases (CVD) [4,5].

Isorhamnetin (3-methyl quercetin, molecular formula: C_16_H_12_O_7_) is a methylated flavonol present in leaves, flowers, and fruits of many plants [5,6,7]. This is the case of *Phaseolus vulgaris* L., belonging to the group of legume plants consumed in the five continents as an essential component of the diet [8]. These seeds have beneficial health properties due to the presence of polyphenolic compounds [9], of which flavonoids stand out, specifically isorhamnetin and its derivatives as isorhamnetin-3-glucoside [10] and isorhamnetin 3-glucuronide [11].

Epidemiological and clinical studies have shown that beans’ consumption is inversely related to coronary artery diseases and the risk of cardiovascular events [12,13]. Various works showed that consuming beans four or more times per week reduces risks of coronary artery disease and CVD [9,13]. In addition, *Phaseolus vulgaris* L. has been related to the inhibition of thrombotic events. Even bean extracts reduced platelet aggregation stimulated by adenosine 5′-diphosphate (ADP) and arachidonic acid. The antiplatelet mechanism was related to activation of protein kinase B (AKT), which decreases the activation of platelets [8].

Isorhamnetin has been used in traditional medicine to prevent and treat various diseases [14,15] due to its cardiovascular, anti-inflammatory, antitumor, antioxidant, antibacterial, antiviral, and anticoagulant activities [5,6,16]. The pharmacological effects of this flavonoid have been reported to be related to the regulation of activated B cell kappa light chain enhancer nuclear factor (NF-κB), PI3K/AKT, mitogen-activated protein kinases (MAPK), and other downstream signaling pathways [6].

In the context of cardioprotective potential, isorhamnetin protects against cardiac hypertrophy by blocking the PI3K-AKT pathway [5,15]. The effects on atherosclerosis in vitro and in vivo have also been evaluated [17]. Isorhamnetin was shown to inhibit atherosclerotic plaque development by activation of PI3K/AKT [17]. In addition, this compound reduces myocardial hypertrophy and fibrosis caused by pressure loading [6,15].

A causal factor in cardiovascular disorders is platelet activation. Platelets play a fundamental role in thrombus formation, atherogenesis, and atherosclerotic lesions progression [18]. The inhibitions of platelet activation by natural products have been described as a central target to prevent thrombus formation [19,20]. Isorhamnetin can inhibit platelet aggregation, but the specific mechanisms have not yet been verified [6]. Consequently, our objective was to evaluate isorhamnetin’s antiplatelet activity against different agonists and explore the specific mechanisms involved in the antiplatelet effects to provide further evidence of its healthy properties.

## 2. Materials and Methods

### 2.1. Chemicals

Thrombin 6 receptor activating peptide (TRAP-6), collagen, and phorbol myristate acetate (PMA), were obtained from Sigma-Aldrich (St. Louis, MO, USA). Antimycin (AA), citrate-dextrose solution, mepacrine, dihydroethidium (DHE), intracellular calcium fluorescence indicator (Fluo-3-AM), and trifluoromethoxyphenylhydrazone (FCCP) were also obtained from Sigma-Aldrich (St. Louis, MO, USA). FITC annexin V apoptosis, PE mouse IgG1 isotype control, and FITC mouse anti-human CD61 were obtained from BD Biosciences (San Diego, CA, USA). Isorhamnetin was obtained from Cayman Chemical, USA. All assays incorporated as vehicle control dimethyl sulfoxide (DMSO) 0.2%.

### 2.2. Preparation of Human Platelets

Platelets were obtained from six healthy volunteers (men and women) who did not consume medication for two weeks. Donors signed the informed consent according to the protocol approved by the Scientific Ethics Committee of the University of Talca (protocol no. 19/2018), following the Declaration of Helsinki [21]. Blood was collected with acid-citrate-dextrose (ACD) 4:1 *v*/*v* and then centrifuged at room temperature for 10 min at 240× *g* to obtain platelet-rich plasma (PRP). PRP was centrifuged (800× *g*) at room temperature for 8 min [22]. Then platelet pellet was resuspended in calcium-free Tyrode’s buffer: ACD (5:1 *v*/*v*). The platelets were washed again by centrifugation at 800× *g* for 8 min [23]. The platelet count was performed on a hematology counter (Mindray BC-3000 Plus hematology counter, Shenzhen, China). Washed platelets were added to an Eppendorf, and anti-CD61-FITC was added. Thus, platelet purity (>99%) was confirmed by flow cytometry Accuri C6 (BD, Biosciences, San Jose, CA, USA) using an anti-CD61-FITC antibody (Supplementary Figure S1). Platelet populations were selected based on cell size using scatter (FSC) versus side scatter (SSC) and CD61 positivity to distinguish it from electronic noise, as described by other authors [22]. Flow cytometry experiments were controlled with PE mouse IgG1 isotype control [24].

### 2.3. Cytotoxic Activity

Washed platelets (3 × 10^8^ platelets/mL) were incubated with isorhamnetin (10, 50 and 100 µM) for 10 min at 37 °C. Subsequently, platelets were centrifuged (800× *g*) for 8 min, and the supernatant was analyzed with the lactate dehydrogenase (LDH) cytotoxicity assay kit (Cayman Chemical, MI, USA). A microplate reader (Thermo Scientific Multiskan Go Microplate Reader, Vantaa, Finland) was used to measure the reaction’s absorbance at 490 nm. The positive control was Triton X-100 (10%) [23].

### 2.4. Platelet Aggregation Assay

Inhibition of platelet aggregation was evaluated by a turbidimetric method using a lumi-aggregometer (Chrono-Log, Havertown, PA, USA) [25,26]. Washed platelets (3 × 10^8^ platelets/mL) were incubated for 5 min with CaCl_2_ (2 mM) plus isorhamnetin (1, 10, 20, 50 and 100 µM) or vehicle (DMSO, 0.2%). Similar concentrations in vitro have been evaluated in other studies [6,27]. Platelet aggregation was induced by TRAP-6 (5 µM), collagen (1 mg/mL), and PMA (100 nM). Platelet aggregation (transmittance) was measured for 6 min [23]. The platelet aggregation percentage was obtained with the AGGRO/LINK software (Chrono-Log, Havertown, PA, USA). Platelet inhibition was calculated as: inhibition of platelet aggregation (%) = 100 − ((platelet aggregation of isorhamnetin/platelet aggregation of negative control) × 100) [28]. The concentration necessary to reduce platelet aggregation by 50% (IC_50_) was obtained from isorhamnetin’s concentration curves (1, 10, 20, 50, and 100 µM).

### 2.5. Phosphatidylserine Externalization

Externalization of phosphatidylserine (PS) in platelets was determined by flow cytometry, following the methodology described by Mendez et al. 2020, with slight modifications [23,29]. Washed platelets (3 × 10^8^ platelets/mL) were incubated for 5 min with CaCl_2_ (2 mM) and subsequently with isorhamnetin (1, 10, and 20 µM) for 10 min at 37 °C. In addition, PS was induced in activated conditions by collagen (1 mg/mL)/TRAP-6 (5 µM). The samples were then diluted with annexin V-binding buffer and pre-incubated in the dark with annexin V-FITC and anti-CD61-PE for 30 min. Samples were examined on an Accuri C6 flow cytometer (BD, Biosciences, San Jose, CA, USA).

### 2.6. Platelet Secretion (Extracellular ATP)

Extracellular adenosine triphosphate (ATP) was determined using Chrono-lume (Chrono-Log, Havertown, PA, USA) [22]. Washed platelets (3 × 10^8^ platelets/mL) were incubated for 5 min with CaCl_2_ (2 mM) and isorhamnetin (1, 10 and 20 µM). The luciferin (Chrono-Lume) reagent and the platelet agonists (TRAP-6 5 µM and collagen 1 µg/mL) were added consecutively. ATP secretion (100%) was obtained with the vehicle (DMSO 0.2%) plus the platelet agonists. Adenosine (10 µM) was used as a positive control of inhibition.

### 2.7. Intracellular Calcium Levels

Washed platelets (3 × 10^8^ platelets/mL) were incubated with Fluo-3-AM (0.4 µM) at room temperature for 30 min as previously described by Mendez et al. 2020 [23]. Subsequently, platelets were diluted (5 × 10^7^ platelets/mL) with Tyrode’s buffer without calcium, and the samples were incubated with the negative control, FCCP (1 µM) or isorhamnetin (1, 10 and 20 µM) for 10 min at 37 °C. The effect of isorhamnetin on the intracellular calcium levels was calculated relative to the vehicle (DMSO 0.2%) using the Accuri C6 flow cytometer (BD, Biosciences, San Jose, CA, USA).

### 2.8. Reactive Oxygen Species

Reactive oxygen species production (ROS) was determined in washed platelets (5 × 10^7^ platelets/mL) with DHE (10 µM) in the presence of isorhamnetin (1, 10, and 20 µM) and CaCl_2_ (2 mM) for 30 min at 37 °C. Antimycin 10 µM was a positive control. ROS formation was analyzed by Accuri C6 flow cytometer (BD, Biosciences, San Jose, CA, USA) [23,30].

### 2.9. Mitochondrial Membrane Potential

Mitochondrial membrane potential (Δψm) was assessed with the permeating cellular dye tetramethylrhodamine methyl ester perchlorate (TMRM) as before described [23]. CaCl_2_ (2 mM) was added to the washed platelets (5 × 10^7^ platelets/mL). Then, TMRM (100 nM) was added and incubated with DMSO 0.2%, isorhamnetin (1, 10, and 20 µM), or FCCP 1 µM at 37 °C for 30 min. The samples were studied in the Accuri C6 flow cytometer (BD, Biosciences, San Jose, CA, USA) [31].

### 2.10. Thrombosis under Flow Conditions on a Damaged Vascular Wall: The Badimon Perfusion Chamber

The effect of isorhamnetin (20 µM) on platelet adhesion and thrombus formation was evaluated under controlled blood flow conditions in the Badimon Perfusion Chamber [32,33]. The procedures used were reviewed and approved by the Institutional Committees for Animal Use and Care (CEEA-IR) and authorized by the Animal Experimental Committee of the local government (#5601) following Spanish law (RD 53/2013) and European Directive 2010/63/EU. The research conforms to the Guide for the Care and Use of Laboratory Animals published by the US National Institutes of Health (NIH Publication no. 85–23, revised 1985) and follows the ARRIVE guidelines [34]. Pig aorta specimens fresh were obtained from a local slaughterhouse. The sample is immediately washed in PBS, cleaned of the adventitia, cut into long pieces, and then frozen at −80 °C until needed. The aortas were thawed in phosphate-buffered saline at 4 °C, open longitudinally, and cut into 30 × 10 mm segments. Aorta substrates were stripped of the intimal layer to expose the underlying thrombogenic medial layer and were mounted in the previously characterized Badimon perfusion chamber. Chambers were placed in series, each containing a piece of the porcine aorta. Porcine arterial blood was collected from healthy untreated animals in citrate-dextrose solution. Platelets were rendered fluorescent by the addition of mepacrine 20 µM. Blood was perfused over the damaged arterial wall place in the Badimon perfusion chamber. Aorta segments were washed with saline buffer and scrapped to recover adherent platelets. Platelets were resuspended in saline buffer (500 µL) and frozen until use. Finally, platelet lysate fluorescence was measured in a Wallac 1420 Victor2 microplate reader (PerkinElmer). Platelet number was calculated from a standard curve (n° platelets vs. fluorescence; 2-fold platelet dilution series), plotting the mean absorbance for each standard against the platelet number on the *y*-axis. The data were linearized, and regression analysis was calculated (*y* = 15,114 x; r^2^ = 0.85).

### 2.11. Statistical Analysis

Data were represented as the mean ± standard error of the mean (SEM) of the experiments and were analyzed using Prism 6.0 software (GraphPad Inc., San Diego CA, USA). The IC_50_ was calculated from the dose-response curves of isorhamnetin. Results were examined by analysis of variance (ANOVA) and Tukey’s post hoc test to determine the significant differences between samples [2]. Differences between the two groups were analyzed by Student’s *t*-test. *p* values < 0.05 were considered statistically significant.

## 3. Results

### 3.1. Cytotoxic Activity on Platelets

**Figure 1:**
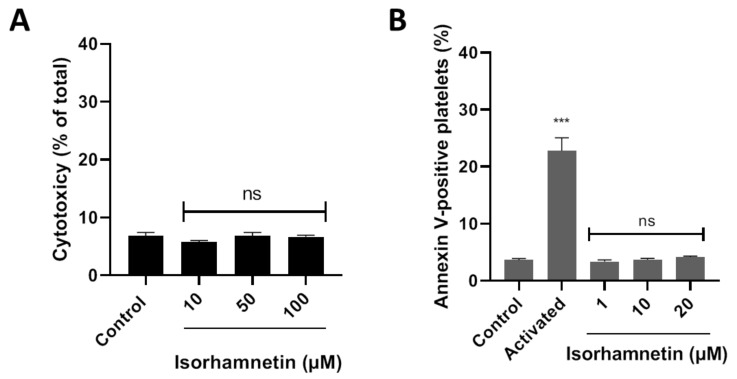
Evaluation of the cytotoxicity of isorhamnetin in washed platelets. (**A**) Effect of isorhamnetin on lactate dehydrogenase (LDH) release. (**B**) Effect of isorhamnetin on PS was evaluated by annexin V-binding in platelets. Activated is collagen/TRAP-6-induced externalization of PS. The results are shown as the mean ± SEM of *n* = 3. Control was DMSO 0.2%. *** *p* < 0.001 vs., control. ns: not significant. PS: phosphatidylserine.

LDH is released from the cell because of damage or lysis. In this context, isorhamnetin’s cytotoxic effects were evaluated at 10, 50, and 100 µM. It was observed that isorhamnetin at none of the concentrations evaluated induced cytotoxic activity (Figure 1A). The PS externalization assay allows us to assess whether the compound under study (isorhamnetin) alters cell viability. We evaluated the levels of PS related to early apoptosis at different concentrations of isorhamnetin (1, 10, and 20 µM). The PS was found to be unchanged in the presence of the isorhamnetin (Figure 1B).

### 3.2. Inhibition of Platelet Aggregation by Isorhamnetin

**Figure 2:**
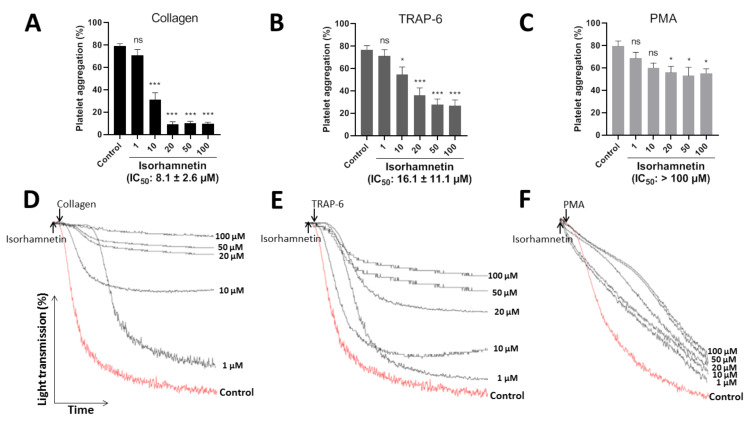
Inhibitory effect of isorhamnetin on platelet aggregation induced by collagen, TRAP-6, and PMA. (**A**) Platelet aggregation induced by collagen. (**B**) Platelet aggregation induced by TRAP-6. (**C**) Platelet aggregation induced by PMA. In this condition, isorhamnetin at 100 µM inhibited PMA-induced platelet aggregation by about 30 ± 6%. (**A**–**C**) The results are shown as the mean ± SEM of *n* = 6. IC_50_ was obtained from dose–response curves. (**D**–**F**) Representative curves of platelet aggregation induced by collagen, TRAP-6, and PMA. * *p* < 0.05, *** *p* < 0.001 and ns (not significant) vs., control (DMSO 0.2%). PMA: phorbol myristate acetate, TRAP-6: thrombin receptor activator peptide 6.

The efficacy of isorhamnetin (1, 10, 20, 50, and 100 µM) to inhibit platelet aggregation was evaluated by turbidimetry stimulated by collagen (1 µg/mL), TRAP-6 (5 µM), and PMA (100 nM) (Figure 2A–C). The representative curves showed the dose-dependent to its platelet antiaggregant activity (Figure 2D–F). Isorhamnetin showed differences in its selectivity to inhibit platelet aggregation induced by platelet agonists. It showed more selectivity when platelets were activated with collagen and TRAP-6. Isorhamnetin (IC_50_ of 8.1 ± 2.6 µM) had a powerful concentration-dependent antiplatelet effect when platelet aggregation was induced by collagen. When TRAP-6 was used as an agonist, the compound inhibited platelet aggregation with IC_50_ of 16.1 ± 11.1 µM. While PMA-induced platelet aggregation had a lower activity (IC_50_ > 100 µM) and at 100 µM inhibited PMA-induced platelet aggregation by about 30 ± 6%. Based on these results, we decided to continue the antiplatelet studies with isorhamnetin 1, 10, and 20 µM.

### 3.3. Isorhamnetin Antiplatelet Mechanism

#### 3.3.1. Platelet Secretion: Extracellular ATP

**Figure 3:**
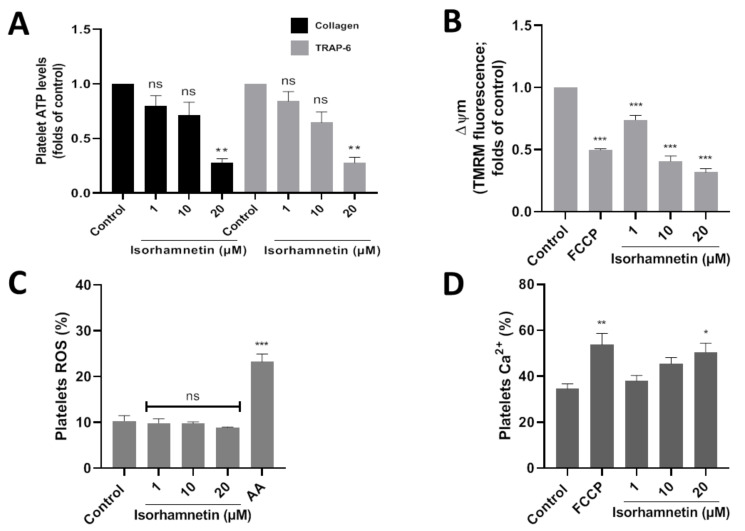
Evaluation of the antiplatelet mechanism of isorhamnetin. (**A**) Platelet ATP secretion stimulated by TRAP-6 or collagen in the presence of isorhamnetin. (**B**) Mitochondrial membrane potential (ΔΨm). (**C**) Levels of platelets ROS. (**D**) Levels of platelets calcium levels. The results are shown as the mean ± SEM of *n* = 3. * *p* < 0.05, ** *p* < 0.01, *** *p* < 0.001 and ns (not-significant) vs., control (DMSO 0.2%). AA: antimycin A 10 µM, ATP: adenosine triphosphate, FCCP: carbonyl cyanide-4- (trifluoromethoxy) phenylhydrazone, ROS: reactive oxygen species, TRAP-6: thrombin receptor activator peptide 6.

Platelet activation is stimulated by platelet secretion products, such as ATP. About the control, isorhamnetin at 20 µM lowers ATP levels to 0.28 ± 0.03 and 0.28 ± 0.05 (folds of control) in collagen- and TRAP-6-stimulated platelet, respectively (Figure 3A). Meanwhile, adenosine (10 µM) about control decreased ATP levels to 0.20 ± 0.05 and 0.19 ± 0.06 (folds of control) in platelets stimulated by collagen and TRAP-6, respectively.

#### 3.3.2. Mitochondrial Membrane Potential

The association between developing CVD and mitochondrial damage is well-known. We study if the isorhamnetin has an antiplatelet activity through the inhibition of mitochondrial bioenergetics. In this context, we observed that isorhamnetin at 1, 10, and 20 µM significantly decreases the membrane potential compared to the control (Figure 3B).

#### 3.3.3. Reactive Oxygen Species (ROS)

Platelet activation is regulated by the endogenous generation of ROS induced by a wide variety of stimuli, for example, at the mitochondrial level by antimycin A. In our study, isorhamnetin was shown not to affect ROS (Figure 3C).

#### 3.3.4. Intracellular Calcium Levels

We assess whether our compound can modulate calcium levels in platelets. Isorhamnetin at 20 µM was shown to increase Ca^2+^ levels relative to baseline (Figure 3D). Isorhamnetin increases the release of calcium from dense granules; this is a response mechanism because the compound has been shown to decrease the membrane potential in the mitochondria.

#### 3.3.5. Thrombosis under Flow Conditions on a Vascular Wall (Badimon Perfusion Chamber)

**Figure 4:**
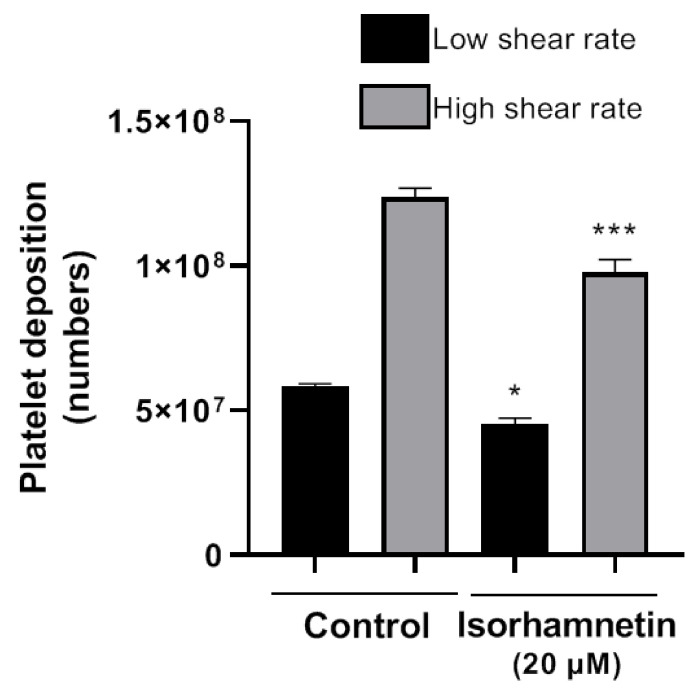
Effect of isorhamnetin on thrombus formation in vitro under low and high shear rate conditions. The results are shown as the mean ± SEM of *n* = 4. * *p* < 0.05 and *** *p* < 0.001 vs., control (DMSO 0.2%).

The Badimon chamber allowed us to evaluate isorhamnetin’s effects on platelet deposition and thrombus formation caused by damaged vessel wall under low and high shear rate conditions. Isorhamnetin induced inhibitory effects at a low shear rate (typical of large arteries, such as the aorta) and at high shear rate (typical of smaller arteries, as the coronaries with stenosis). Isorhamnetin at 20 µM induced a significant reduction in platelet deposition versus an untreated blood sample (Figure 4) at both shear conditions. The data confirm the antithrombotic effect of isorhamnetin on thrombus triggered by a biological vascular substrate and prothrombotic flow conditions mimicking arteries perfused by blood at high shear stress.

## 4. Discussion

Platelets are the smallest blood cells, and their activation at sites of the vascular lesion is essential for thrombus formation [35]. The treatment to prevent thrombosis associated with platelet activation requires the inhibition of platelet activation [36]. Aspirin and clopidogrel are essential in managing platelet aggregation, but several patients continue to suffer from recurrent thrombotic problems and even can induce bleeding [37]. That is why finding potential antiplatelet agents in foodstuff used daily with the diet would represent a clear advantage to reduce the challenge of thrombotic risk. In this context, it is vital to promote adequate nutrition in the population by consuming fruits and vegetables, to achieve a healthy lifestyle [8]. Different investigations show that the intake of flavonoids present in vegetables and fruits can reduce CVD, modulating platelet activation [18].

Isorhamnetin has broad pharmacological effects, such as anti-osteoporosis [38], anti-inflammatory [39], antioxidants [40], anti-apoptosis [17], immune regulatory [41], and other pharmacological effects for which the mechanisms of action have been described. Therefore, in this article, we have investigated its antiplatelet potential and the mechanisms involved, contributing to generating the evidence necessary for future in vivo studies.

LDH activity can be used as an indicator of cell membrane integrity and, therefore, a measure of cytotoxicity [35]. Isorhamnetin cytotoxicity levels were evaluated by measuring cytosolic LDH release without observing any platelet damage. In addition, isorhamnetin did not affect PS exposure, a modification in platelets associated with mitochondrial apoptotic-like events [42,43], confirming non-cytotoxic effects on platelets. We used light transmission aggregometry to evaluate the inhibitory activity of isorhamnetin (1 to 100 µM) in platelet aggregation induced by collagen, TRAP-6, and PMA. Similar concentrations have been studied to evaluate isorhamnetin’s protective effect and its derivatives [27,44,45,46].

Platelet agonists activate different pathways in signal transductions. TRAP-6 acts as a PAR1 agonist in platelet activation [35,47]; glycoprotein VI (GPVI) is the primary collagen receptor in platelet activation. On the other hand, integrin α2β1 and CD36 bind directly to collagen, while subunits of the GP Ib-IX complex (GP) Ibα and integrin αIIbβ3 interact with von Willebrand factor (vWF) bound to collagen [35,48]. Isorhamnetin inhibited, in a dose-dependent manner, platelet aggregation induced by TRAP-6 and collagen. Our results suggest that isorhamnetin inhibited the platelet GPVI-mediated signaling pathway [42,49]. Ingestion of isorhamnetin-rich onion soup has been reported to inhibit collagen-stimulated platelet aggregation [6,50]. Conversely, isorhamnetin did not inhibit platelet aggregation stimulated by protein kinase C (PKC) agonist as PMA, suggesting that isorhamnetin acts upstream of PKC. Compounds, such as amyrins, have been reported to disrupt MAPK and arachidonic acid metabolisms, related to inhibition of upstream PKC targets [51,52].

Mitochondria via ATP production are involved as central drivers in platelet activation. In this context, inhibition of mitochondrial function has been reported as an antiplatelet target via a decrease of ATP levels [53,54]. We decided to evaluate whether isorhamnetin has antiplatelet activity by inhibiting mitochondrial function. This compound at 20 µM causes a decrease of membrane potential without affecting ROS levels and decreasing ATP levels. In addition, due to isorhamnetin’s action on the mitochondria, an increase in intraplatelet calcium levels was observed. These results show that isorhamnetin decreased platelet activation via inhibition of mitochondrial function, which is associated with potent non-selective inhibition of platelet aggregation induced by TRAP-6 and collagen.

It has been described that atherothrombosis is initiated by collagen exposure when endothelial damage occurs, and this triggering platelet activation/adhesion [42,55]. In this context, we investigated the effects of isorhamnetin on thrombosis on vascular damage under flow conditions. We showed that this compound inhibited thrombus formation triggered by exposure of a highly thrombogenic surface to flowing blood (Badimon chamber).

Although this study has some limitations since the effects of isorhamnetin on platelet aggregation are more complex in vivo conditions, our results support scientific evidence that isorhamnetin helps reduce platelet activation and thereby thrombotic and cardiovascular risk.

## 5. Conclusions

Diet and nutrition influence the progression and/or treatment of CVD. Isorhamnetin may be a promising scaffold compound to develop new antiplatelet agents with specific action on thrombotic diseases. Isorhamnetin did not induce cytotoxic effects. Its antiplatelet potential was significant in collagen-stimulated platelet aggregation. Consecutively, it induced a fall in membrane potential and a decrease in ATP levels. The evidence presented in this work suggests that this flavonoid may play an important role in health maintenance and possibly protect against cardiovascular disease associated with the inhibition of platelet function and a possible reduction in the risk of thrombosis. Certainly, future studies, such as absorption and metabolism, are needed to translate the antiplatelet activity of in vitro dose–response to in vivo concentrations (human).

## Data Availability

Data is contained within the article and supplementary material.

## Supplementary Materials

The following are available online at https://www.mdpi.com/article/10.3390/antiox10050666/s1, Figure S1: Representative dot plots of CD61+ (platelets).

## Abbreviations

AA	Antimycin
ADP	Adenosine diphosphate
ACD	Acid-citrate-dextrose
AKT	Protein kinase B
ANOVA	Analysis of variance
ATP	Adenosine triphosphate
CDV	Cardiovascular diseases
DHE	Dihydroethidium
DMSO	Dimethyl sulfoxide
ERK ½	Extracellular signal-regulated kinases 1/2
FCCP	Carbonylcyanide p-trifluoromethoxyphenylhydrazone
FITC	Fluorescein-5-isothiocyanate
Fluo-3-AM	Intracellular calcium fluorescence indicator
FSC	Forward scatter
GP Ibα	Subunits of the GP Ib-IX complex
GPVI	Glycoprotein VI or glycoprotein receptor for collagen
IC_50_	Half-maximum inhibitory concentration
LDH	Lactate dehydrogenase
MAPK	Mitogen-activated protein kinases
Δψm	Mitochondrial membrane potential
NF-κB	Activated B cell kappa light chain enhancer nuclear factor
PAR1	Protease-activated receptor-1
PI3K	Phosphatidylinositol-3-kinase
PKC	Protein kinase C
PMA	Phorbol myristate acetate
PRP	Platelet-rich plasma
PS	Phosphatidylserine
ROS	Reactive oxygen species
SEM	Standard error of the mean
SSC	Side scatter
TMRM	Tetramethylrhodamine methyl ester perchlorate
TRAP-6	Thrombin receptor activator peptide 6
vWF	Von Willebrand factor
