# Supplementary material for: Antiplatelet Activity of Isorhamnetin via Mitochondrial Regulation
Source: Antioxidants (Basel). 2021 Apr 25;10(5):666. doi: 10.3390/antiox10050666 (PMC8146847; doi:10.3390/antiox10050666)
Supplement: Supplementary file 1 [file antioxidants-10-00666-s001.zip › antioxidants-1169160-supplementary.pdf]

Supplementary Figure

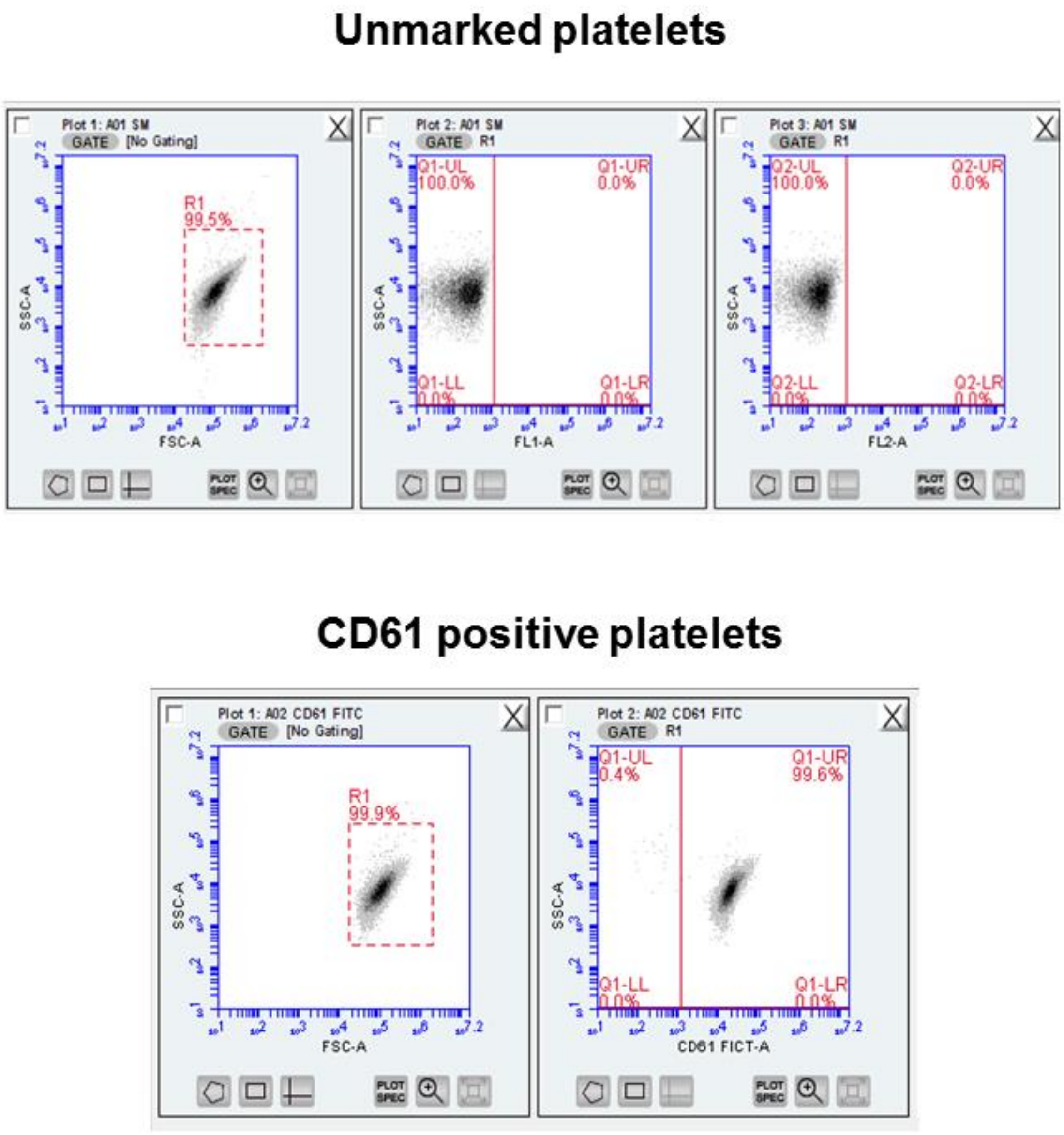

Supplementary Figure 1. Representative dot plots of CD61+ (platelets).
